# Possible involvement of altered expression of BDNF exon II gene and specific dopamine receptors in simvastatin induced beneficial effects in depression

**DOI:** 10.1186/1755-8166-7-S1-P67

**Published:** 2014-01-21

**Authors:** Digvijay G Rana, Amrut Patel, CG Joshi, Ramesh K Goyal

**Affiliations:** 1 Department of Pharmacology, Sigma Institute of Pharmacy, Vadodara, India; 2 Department of Biotechnology, Anand Veterinary College, Anand, India; 3 Institute of Life Sciences, Ahmedabad University, Ahmedabad, Gujarat, India

**Keywords:** BDNF exon II gene, dopamine, statins, depression

## Background

The up regulation of central BDNF gene expression has been suggested in the treatment of major depression. Chronic administration of dopaminergic agents activates the function of CREB which results into the up regulation of the BDNF gene expression. Statin therapy is associated with a reduced risk of depression and could be of therapeutic potential for major depression.

## Materials and methods

We have examined a possible link amongst simvastatin, bromocriptine, haloperidol and levodopa in accordance with BDNF exon II gene expression using RT-PCR method in mice treated with standard paradigm of chronic mild stress procedure for 14 days. We specifically determined if the oral administration of simvastatin would affect the efficacy of bromocriptine, haloperidol or levodopa in mediating the regulation of the BDNF exon II gene expression.

## Results

The results of RT-PCR method revealed the differential expression patterns for the expression of BDNF exon II gene in brain of mouse by indicating the three different bands, as evidenced previously to be the three different exon II transcript variants in mouse namely BDNF IIA, BDNF IIB and BDNF IIC. Mice treated with bromocriptine or levodopa in combination with simvastatin for 14 days could synergize the up regulation of for the expression of specific BDNF exon II transcript as compared to simvastatin alone whereas the mice treated with haloperidol in combination with simvastatin for 14 days could abolish the for the expression of up regulation of specific BDNF exon II transcript compared to simvastatin alone.

## Conclusion

The results of the above study suggests linkage between the function of dopamine or dopamine D_2_ like receptor and differential expression patterns for the expression of BDNF exon II gene in brain of mouse which further strengthen the emerging hypothesis, suggesting the ability of neuronal systems to exhibit the appropriate adaptive plasticity could contribute to the treatment of depression. Further, the dopaminergic agents in accordance with the cholesterol lowering drug as adjuncts may reduce the depressive like behavior more significantly and facilitation of antidepressant action of dopaminergic agents may be correlated with HMGR or cholesterol or mevalonate pathway.

